# Evaluation of Real-World Vancomycin Dosing and Attainment of Therapeutic Drug Monitoring Targets

**DOI:** 10.3390/pharmacy11030095

**Published:** 2023-06-06

**Authors:** Nicole Bradley, Kimberly Ng

**Affiliations:** College of Pharmacy and Health Sciences, St. John’s University, Queens, NY 11439, USA; ngk3@stjohns.edu

**Keywords:** vancomycin, pharmacokinetics, therapeutic drug monitoring, AUC/MIC ratio

## Abstract

In 2020, the Infectious Diseases Society of America (IDSA) recommended a change in vancomycin therapeutic drug monitoring from trough-based to AUC/MIC-based to optimize vancomycin’s efficacy and reduce nephrotoxicity. Many hospitals have not implemented this change due to barriers such as the cost of AUC/MIC software and lack of provider familiarity. The purpose of this study was to determine the rate of AUC/MIC ratio target attainment using current trough-based vancomycin dosing practices at a city hospital. The rates of acute kidney injury (AKI) were also evaluated. Vancomycin orders were reviewed retrospectively to determine the expected AUC/MIC ratios using first-order pharmacokinetic equations over a 7-month period. Orders were excluded if they were written for a one-time dose, for individuals less than 18 years of age, or for those on hemodialysis. A total of 305 vancomycin orders were included in this review. Overall, 27.9% (85/305) of vancomycin orders attained the AUC/MIC ratio target of 400–600 mg·h/L as recommended by the guidelines. Nearly 35% (106/305) achieved AUC/MIC ratios below 400 mg·h/L and 37.4% (114/305) achieved AUC/MIC ratios above 600 mg·h/L. Orders for obese patients were significantly more likely to have below the target AUC/MIC ratios (68% vs. 23.9%, X^2^ 48.48, *p* < 0.00001) and non-obese patients were significantly more likely to have above the target AUC/MIC ratios (45.7% vs. 12%, X^2^ 27.36, *p* < 0.00001). The overall rate of acute kidney injury observed was 2.6%. Most vancomycin orders did not attain therapeutic drug monitoring targets, reflecting the ongoing clinical challenge of optimizing vancomycin doses and implementing new guideline recommendations.

## 1. Introduction

Vancomycin is a glycopeptide antibiotic indicated for the treatment of Gram-positive bacteria, including *Streptococci*, *Enterococci*, and methicillin-resistant *Staphylococcus aureus* (MRSA) [1]. It inhibits the polymerization of peptidoglycans in the bacterial cell wall by binding to D-alanyl D-alanine, ultimately causing cell death. Vancomycin displays an area-under-the-curve-to-minimum-inhibitory-concentration ratio (AUC/MIC) killing activity, and the initial dosing recommendations are dependent on factors such as indication, renal function, and body weight. Adverse events associated with intravenous vancomycin include nephrotoxicity and vancomycin infusion reactions. To prevent vancomycin-associated nephrotoxicity and optimize efficacy, therapeutic drug monitoring (TDM) is required for vancomycin [1]. Supratherapeutic dosing can result in adverse effects while subtherapeutic dosing can negatively impact patient outcomes if an infection is not properly managed.

In 2009, practice guidelines for vancomycin TDM for the treatment of *S. aureus* infection were initially published. These original guidelines recommended using vancomycin trough concentrations as a surrogate marker for the AUC/MIC ratio for ease of use in clinical practice, targeting trough levels between 10 and 20 mg/L, depending on the infection type [2]. In 2020, a revised consensus guideline was released, recommending a change in vancomycin TDM from trough-based to AUC/MIC-based, targeting levels of 400–600 mg·h/L, to reduce the rates of nephrotoxicity associated with trough-only monitoring practices. Utilization of Bayesian software programs is the preferred method of AUC/MIC monitoring recommended by the guidelines; however, first-order pharmacokinetic equations (PK) may be used as an alternative. The guidelines also highlight dosing and monitoring considerations in special populations such as obesity, renal disease, and pediatric patients [3]. Despite this updated guidance, the implementation of vancomycin AUC/MIC-based dosing and monitoring has been limited in the United States (U.S.). In 2021, a survey of approximately 200 hospital pharmacists found that a majority had not yet implemented AUC dosing. Common barriers cited were the cost of software programs and a lack of provider familiarity [4].

Like other institutions across the U.S., our 545-bed city hospital has not yet implemented vancomycin AUC/MIC dosing and monitoring, and trough-based monitoring remains the standard for patients receiving vancomycin. Medical residents are responsible for placing orders for trough-based monitoring, and no standardized pharmacy-based protocol for dosing or monitoring vancomycin exists. Antimicrobial stewardship (AMS) services were initiated in 2017 when the new Joint Commission standard for medication management addressing antimicrobial stewardship became effective, and they have since expanded [5]. AMS services are provided by pharmacy faculty preceptors and Advanced Pharmacy Practice Experience (APPE) students at the hospital in select units and include vancomycin TDM. With the vancomycin guideline updates, faculty members worked to develop a mechanism to evaluate current vancomycin dosing practices in attaining TDM targets.

The objective of this study is to determine the rate of AUC/MIC ratio target attainment using current trough-based vancomycin dosing practices. Rates of acute kidney injury (AKI) were also evaluated.

## 2. Materials and Methods

Vancomycin orders were reviewed retrospectively to determine the expected AUC/MIC ratios using first-order pharmacokinetic equations (Table 1) over a 7-month period to determine the rate of AUC/MIC ratio target attainment. Vancomycin serum concentrations that were drawn at steady state were utilized. If only one vancomycin level was available, the elimination rate constant was estimated using the Matzke Equation, and the subsequent level was extrapolated using the first-order pharmacokinetic equations described in Table 1. When multiple vancomycin peak and trough levels were available, the earliest drawn levels at steady state were used to calculate the AUC/MIC ratio. Vancomycin serum concentrations were determined by the clinical laboratory using standard practice laboratory techniques. Orders were excluded if they were written for a one-time dose, for individuals less than 18 years of age, those with no vancomycin level available, or patients on hemodialysis. Baseline demographic information including sex, age, weight, and baseline renal function was collected. The baseline renal function was determined using the Cockcroft–Gault Equation. Outcomes were then stratified based on obesity status and by renal function. Obesity was defined as a body mass index (BMI) of at least 30 kg/m^2^, and AKI as an increase in serum creatinine (SCr) of at least 0.5 mg/dL or a 50% increase from baseline on 2 consecutive days in the absence of alternative explanation, in accordance with the vancomycin TDM guidelines [3]. Results were analyzed using descriptive statistics. The Chi-Square test and Fisher Exact Test were used to compare categorical data.

## 3. Results

Three hundred and five vancomycin orders were included in this review. As seen in Table 2, the majority of orders were for males (75.4%) with a mean age of 53.4 years and mean weight of 74.6 kg. Almost 25% (75/305) of orders were for obese patients and 77.7% (237/305) for patients with a creatinine clearance (CrCl) of at least 60 mL/min. Approximately 65% of vancomycin orders were for complicated infections (central nervous system, pneumonia, bone and joint, bloodstream infections, or sepsis), 28.8% for uncomplicated infection (urinary tract infection or skin and soft tissue infection), and 6.2% for unknown indications. The average dose of vancomycin ordered was 16.3 mg/kg/dose. All *Staphylococcus aureus* isolates had vancomycin MIC values of 1 or less. If no culture and sensitivity information was available, an assumption of a vancomycin MIC value of 1 or less was made.

**Figure 1:**
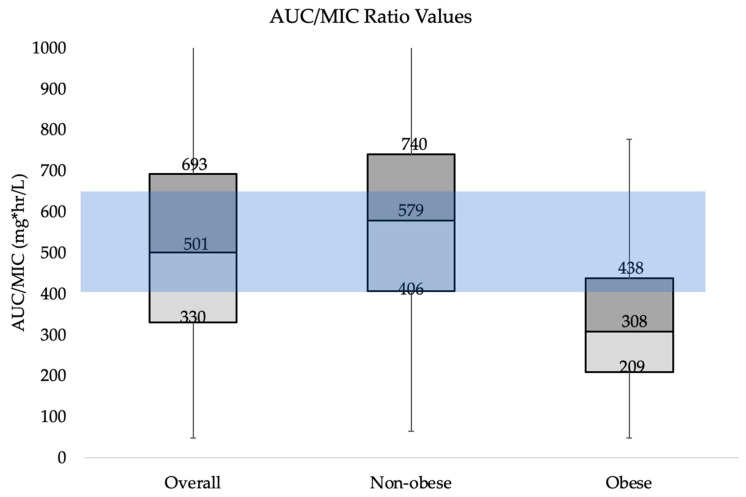
Box and Whisker plot of AUC/MIC ratio attained for subject include. *AUC*/*MIC* ration of 400–600 mg·h/L is recommended.

Overall, only 27.9% (85/305) of vancomycin orders attained the AUC/MIC ratio target of 400–600 mg·h/L as recommended by the guidelines. Nearly 35% (106/305) achieved AUC/MIC ratios below 400 mg·h/L and 37.4% (114/305) achieved AUC/MIC ratios above 600 mg·h/L (Table 3). Orders for obese patients were significantly more likely to have below-goal AUC/MIC ratios (68% vs. 23.9%, X^2^ 48.48, *p* < 0.00001) and non-obese patients were significantly more likely to have above-goal AUC/MIC ratios (45.7% vs. 12%, X^2^ 27.36, *p* < 0.00001) (Figure 1).

All orders for patients with CrCl < 30 mL/min (100%, 19/19) achieved above-goal AUC/MIC ratios and were significantly less likely to attain the target ratio compared to orders for patients with CrCl above 30 mL/min (Fisher exact 0.0026, *p* < 0.05).

The overall rate of acute kidney injury observed was 2.6% (8/305). No difference in the likelihood of AKI based on obesity status or baseline renal function was observed. Of note, initial AUC calculations were conducted prior to AKI development in these eight patients.

## 4. Discussion

Our study demonstrates that real-world dosing of vancomycin frequently did not result in an AUC/MIC ratio target attainment of 400–600 mg·h/L as recommended by the therapeutic drug monitoring guidelines. The lack of target attainment is particularly concerning as clinical outcome data have demonstrated that early vancomycin target attainment is associated with decreased mortality rates in diseases such as MRSA bacteremia [9]. Additionally, vancomycin AUC/MIC ratio values above 600 mg·h/L are associated with an increased risk of AKI [10,11]. Consequences of AUC/MIC values less than 400 mg·h/L may include suboptimal killing activity against MRSA and potential vancomycin resistance emergence. With approximately a third of vancomycin orders achieving above-goal AUC/MIC ratios, and a third achieving below-goal AUC/MIC ratios, current vancomycin dosing practices do not optimize vancomycin killing activity, increase the risk of nephrotoxicity, and have the potential to select for bacterial resistance.

Moreover, the vancomycin therapeutic drug monitoring guidelines have specific recommendations in special patient populations where vancomycin displays unique pharmacokinetic parameters that make dosing and monitoring more challenging. These special populations include obese patients and patients with baseline renal dysfunction [3]. Our study revealed inadequate vancomycin dosing in both these special populations. For example, obese patients were frequently underdosed and were more likely to have below-goal AUC/MIC ratios. The mean vancomycin dose received among obese patients was 14.4 mg/kg/dose, which is below the 15–20 mg/kg/dose traditionally recommended [3]. The lower-than-recommended vancomycin doses that were utilized in obese patients compared to non-obese patients confounds our ability to determine if obese patients were more frequently subtherapeutic because of issues with initial dosing versus differences in body composition. Additionally, compared to the non-obese group, the obese patients included were younger and had better baseline renal function. Therefore, it may be expected that these patients cleared vancomycin more quickly, leading to more frequent subtherapeutic levels. Past studies also describe obese patients as frequently underdosed, with one study finding that only 0.6% of obese patients received appropriate vancomycin doses [12]. When considering patients with baseline renal impairment, all patients with CrCl < 30 mL/min achieved above-goal AUC/MIC ratios, despite vancomycin doses being renally adjusted. Additionally, these patients were more likely to have above-goal AUC/MIC ratios compared to those with higher baseline CrCl. This finding is worrisome as higher AUC/MIC ratios increase the risk of nephrotoxicity, which may be further propagated by baseline renal impairment. Real-world vancomycin dosing in these special populations highlights the inadequacy of current dosing practices and the need for careful pharmacokinetic dosing and monitoring.

Our findings are consistent with previously published studies. For example, a recent study by Van der Haggen and colleagues also found low rates of AUC/MIC ratio target attainment with current vancomycin dosing practices. The study included 150 patients ranging from neonates to adults. Subtherapeutic levels were found in 68% of adult vancomycin orders, 76% of children, and 52% of neonates [13]. Another study evaluated vancomycin prescribing practices and their correlation to TDM guidelines in 163 patients. The study found that only 24% of patients received a vancomycin loading dose, 72% of which were lower than recommended. When vancomycin levels were available, dose adjustments were not made for 60% of subtherapeutic values and 43% of supratherapeutic values. The authors concluded that there was poor compliance with institutional vancomycin TDM guidance, despite provider awareness of guidelines [14].

While overall rates of vancomycin-associated AKI were low in our study, it is important to note that no evaluation of concomitant nephrotoxic agent, cumulative vancomycin dose, or duration was conducted. Each of these factors is known to increase the risk of vancomycin-induced AKI [3]. A previous meta-analysis cites the prevalence of vancomycin-induced AKI ranging from 5% to 43% depending on various factors [15].

The lack of target attainment seen with current vancomycin dosing and monitoring practices provides an opportunity for improvement. Previous studies have demonstrated that pharmacist-driven vancomycin dosing and monitoring can have beneficial outcomes such as improvements in target attainment, reduction in vancomycin-induced AKI, and lower rates of mortality [16,17]. A recent meta-analysis was conducted to determine the relationship between pharmacist intervention and vancomycin outcomes. The meta-analysis included 34 studies and almost 20,000 participants and found that pharmacist intervention in vancomycin management resulted in significantly lower rates of AKI (OR 0.52, 95% CI; [0.41, 0.67], *p* < 0.00001), significantly higher rates of target attainment (OR 2.86, 95% CI [2.23, 3.67], *p* < 0.00001), and significantly lower rates of mortality (OR 0.47, 95% CI [0.31, 0.72], *p* = 0.0004) [16]. Another retrospective cohort study evaluated rates of monitoring compliance after a pharmacist-driven vancomycin monitoring initiative was implemented. The study found a significant improvement in the number of patients with appropriate vancomycin trough level monitoring (27.3% vs. 55.8%, *p* value < 0.001) in the post-implementation cohort and a decrease in the number of patients without vancomycin level monitoring (30% vs. 7.6%, *p* value < 0.001) [17].

Limitations of this study include AUC/MIC estimates made using first-order pharmacokinetic equations and our need to extrapolate vancomycin peak levels. The utilization of Bayesian software would allow for more precise AUC/MIC estimates [3]. Free Bayesian software is available through websites such as Clin-calc.com and GlobalRPH.com; however, these calculators may not be appropriate for all patient populations [18,19]. Like many institutions across the U.S. [4], our hospital does not utilize Bayesian software.

## 5. Conclusions

Most vancomycin orders did not attain the therapeutic drug monitoring targets recommended by guidelines to improve vancomycin activity and reduce toxicity. Our study reflects the ongoing clinical challenge of optimizing vancomycin dosing and monitoring and transitioning to an AUC/MIC-guided approach. It highlights the need for improved vancomycin dosing and monitoring protocols and opportunities for pharmacist-driven vancomycin dosing. Prospective implementation of AUC/MIC ratio dosing and monitoring would help to improve vancomycin use at our institution.

## Figures and Tables

**Table 1:** First-Order Pharmacokinetic Equations Used [6,7,8].

AUC Calculation When 2 Levels Available		
**Step**	**Description**	**Equation**
1	Calculate observed K_e_ from 2 levels	K_e_ = ln (C_1_/C_2_)/t_1_ − t_2_
2	Calculate true peak (Cmax)	Cmax = C_1_/(e^−Ke(tin)^)
3	Calculate true trough (Cmin)	Cmin = Cmax (e^−ke(tau−tin)^)
4	Calculate AUC during infusion using linear trapezoidal rule	AUC_inf_ = t_in_ (Cmax + Cmin/2)
5	Calculate AUC during elimination using linear trapezoidal rule	AUC_elim_ = (Cmax − Cmin)/K_e_
6	Calculate AUC_24_	AUC_24_ = AUC_inf_ + AUC_elim_ (24/tau)
AUC Calculation when 1 level available		
1	Estimate K_e_ using Matzke Equation	K_e_ = 0.00083 × CrCl + 0.0044
2	Estimate true trough (Cmin)	Cmin = Cmax (e^−ke(tau−tin)^)
3	Extrapolate level needed, (Cmax)	Cmax = Cmin/(e^−Ke(tin)^)
4	Calculate AUC during infusion using linear trapezoidal rule	AUC_inf_ = t_in_ (Cmax + Cmin/2)
5	Calculate AUC during elimination using linear trapezoidal rule	AUC_elim_ = (Cmax − Cmin)/K_e_
6	Calculate AUC_24_	AUC_24_ = AUC_inf_ + AUC_elim_ (24/tau)

K_e_ = elimination rate constant, C_1_ = concentration 1, C_2_ = concentration 2, t = time level was drawn, Cmax = maximum concentration, Cmin = minimum concentration, t_in_ = infusion time, tau = dosing interval. AUC_inf_= Area under the infusion curve, AUC_elim_ = area under the elimination curve, AUC_24_ = Area under the concentration–time curve over 24 h, CrCl = creatinine clearance using Cockcroft gault.

**Table 2:** Baseline Demographics.

	Overall (N = 305)	Non-Obese (N = 230)	Obese (N = 75)
Sex (% Female)	24.6	19.1	41.3
Age (years, mean ± SD)	53.8 ± 18.4	55.1 ± 19	49.8 ± 15.8
Weight (kg, mean ± SD)	74.6 ± 20.5	67.2 ± 15	97.5 ± 22.1
SCr (mg/dL, mean ± SD)	0.93 ± 0.69	0.99 ± 0.77	0.78 ± 0.30
CrCl (mL/min, mean ± SD)	125 ± 91	109 ± 75	175 ± 91
**Vancomycin Considerations**			
Dose (mg/kg/dose, mean ± SD)	16.3 ± 3.9	17.5 ± 10.1	14.4 ± 2.5
Indication (%)			
Uncomplicated (Cellulitis, UTI)	88 (28.8%)	54 (23.5%)	34 (45.3%)
Complicated (CNS, PNA, Bone, BSI, Sepsis)	198 (64.9%)	162 (70.4%)	36 (48%)
Unknown	19 (6.2%)	14 (6.1%)	5 (6.6%)
Vancomycin Levels			
Availability of appropriately drawn trough level (%)	201/305 (65.9)	151/230 (65.7)	50/75 (66.7)
Mean number of serum concentrations per subject	1.7	1.8	1.6

UTI: urinary tract infection, CNS: central nervous system, PNA: pneumonia, BSI: bloodstream infection).

**Table 3:** Rates of AUC/MIC ratio target attainment based on obesity status.

	Overall (N = 305)	Non-Obese (N = 230)	Obese (N = 75)	X^2^
**Therapeutic AUC/MIC Ratio (400–600 mg·h/L)**				
n/N (%)	85/305 (27.9)	70/230 (30.4)	15/75 (20)	3.06, *p* = 0.08
Median AUC (IQR)	495 (117.5)	501 (115.8)	449 (77)	
**Below-Goal AUC/MIC Ratio (<400 mg·h/L)**				
n/N (%)	106/305 (34.8)	55/230 (23.9)	51/75 (68)	48.48, *p* < 0.00001
Median AUC (IQR)	270 (142)	277 (150)	262 (151)	
**Above-Goal AUC/MIC Ratio (>600 mg·h/L)**				
n/N (%)	114/305 (37.4)	105/230 (45.7)	9/75 (12)	27.36, *p* < 0.00001
Median AUC (IQR)	758 (375)	791 (435)	681 (80)	
**Rate of AKI**				
n/N (%)	8/305 (2.6)	4/230 (1.7)	4/75 (5.3)	2.86, *p* = 0.091

## Data Availability

Data supporting the results of this study can be requested via the corresponding author.
